# Accounting for country- and time-specific values in the economic evaluation of health-related projects relevant to low- and middle-income countries

**DOI:** 10.1093/heapol/czab104

**Published:** 2021-08-19

**Authors:** James Lomas, Karl Claxton, Jessica Ochalek

**Affiliations:** Centre for Health Economics, University of York, York YO10 5DD, UK; Centre for Health Economics, University of York, York YO10 5DD, UK; Department of Economics and Related Studies, University of York, York YO10 5DD, UK; Centre for Health Economics, University of York, York YO10 5DD, UK

**Keywords:** Cost-effectiveness analysis, benefit–cost analysis, global health, economic growth, low-income countries, middle-income countries

## Abstract

Economic evaluation of health-related projects requires principles and methods to address the various trade-offs that need to be made between costs and benefits, across sectors and social objectives, and over time. Existing guidelines for economic evaluation in low- and middle-income countries embed implicit assumptions about expected changes in the marginal cost per unit of health produced by the healthcare sector, the consumption value of health and the appropriate discount rates for health and consumption. Separating these evaluation parameters out requires estimates for each country over time, which have hitherto been unavailable. We present a conceptual economic evaluation framework that aims to clarify the distinct roles of these different evaluation parameters in evaluating a health-related project. Estimates for each are obtained for each country and in each time period, based on available empirical evidence. Where existing estimates are not available, for future values of the marginal cost per unit of health produced by the healthcare sector, new estimates are obtained following a practical method for obtaining projected values. The framework is applied to a simple, hypothetical, illustrative example, and the results from our preferred approach are compared against those obtained from other approaches informed by the assumptions implicit within existing guidelines. This exposes the consequences of applying such assumptions, which are not supported by available evidence, in terms of potentially sub-optimal decisions. In general, we find that applying existing guidelines as done in conventional practice likely underestimates the value of health-related projects on account of not allowing for expected growth in the marginal cost per unit of health produced by the healthcare sector.

**Key messages**

- Economic evaluation of health-related projects requires principles and methods to address the various trade-offs that need to be made between costs and benefits, across sectors and social objectives, and over time.
- Conventional practice in health economics is not always clear on how to analyse these trade-offs and often embeds implicit assumptions about expected changes in resource constraints and societal preferences within the discount rates used to evaluate projects.
- One reason for this is that separating these arguments out requires a range of parameter estimates for countries over time that have hitherto been unavailable.
- This paper marshals available evidence to inform estimates of evaluation parameters by country and over time to produce a flexible evaluation framework that can inform decision-makers in a transparent manner.
- Even with a highly stylized simple health-related project, different assumptions about evaluation parameters for each country over time lead to important differences in results for some countries.
- Users of guidelines should think carefully about the appropriateness of implicit assumptions in the context of their own country. Writers of future guidelines should seek to improve the transparency regarding assumptions about evaluation parameters.

## Introduction

Economic evaluation of a health-related project (such as a healthcare intervention or a health technology or programme of care for a particular indication) can be used to support decision-making in low- and middle-income countries (LMICs) (Drummond *et al.*, 2015). It is most commonly operationalized through the application of some form of cost-effectiveness analysis (CEA) (WHO, 2003; 2019; Wilkinson *et al.*, 2016) or, less commonly, benefit–cost analysis (BCA) (Robinson *et al.*, 2019b).

The effectiveness of a health-related project is typically denominated in terms of its impact on a generic measure of health such as a disability-adjusted life year (DALY) or a quality-adjusted life year, but other objectives such as consumption, which comprises both the consumption value of health effects and the consumption of non-health goods and services, may also be considered. The adopted perspective of the analysis determines the appropriate scope of which costs are included (Sanders *et al.*, 2016).

Economic evaluation allows for the calculation of the net benefit of a project, which represents its effect net of the opportunity cost (the value of the best alternative use of the resources required for the project). This depends not only on the choice of objective and perspective but also on the assumed source of the resources. Where the healthcare sector budget available for a project is exogenous to the decision, the resources are obtained from elsewhere within the budget, and the marginal cost per unit of health produced by the healthcare sector ($k$) may be used to calculate the health opportunity cost (Woods *et al.*, 2016; Ochalek *et al.*, 2018). This can be used to quantify the net health benefit, but for comparisons beyond health, the net health benefit may need to be multiplied by the consumption value of health ($v$) in order to compare with the consumption of non-health goods and services (Brouwer *et al.*, 2018).

Both $k$ and $v$ parameters are therefore vital to economic evaluation of this kind, with $k$ used to calculate health opportunity costs and $v$ used to convert health into consumption value, but the separate role for these is not explicitly acknowledged within existing guidelines. Instead, CEA typically refers to a cost-effectiveness threshold against which the incremental cost-effectiveness ratio (ICER) of a project is compared. In guidelines, the basis for a cost-effectiveness threshold is not always explicitly given and could reflect $k$ or $v$ or it may be arbitrary. Historically, an arbitrary cost-effectiveness threshold range of one to three times gross domestic product (GDP) per capita was advised by the World Health Organization (WHO), and its use is still observed in practice in LMICs (Bertram *et al.*, 2016; Leech *et al.*, 2018). More recent guidelines either do not state the basis of the cost-effectiveness threshold (WHO, 2003; 2019) or recommend the use of $k$ (Wilkinson *et al.*, 2016). While guidelines for CEA in LMICs recommend that the choice of cost-effectiveness threshold depends upon the country to which the analysis relates, there is no consideration of how it is likely to evolve over time in each country (resulting in an implicit assumption of constant growth or remaining constant). In contrast, the guideline for BCA in LMICs recommends that health is valued using $v$, with guidance on how this may change over time but acknowledge no role no for $k$ (Robinson *et al.*, 2019b).

Also vital is accounting for the timing of costs and effects. This is conventionally done using discount rates where impacts of a project in future years are downweighted compared to the present, often motivated by the literature on positive time preference. In particular, guidelines have often adopted a constant discount rate of 3% per annum for all countries, which is applied to all costs and effects (Weinstein *et al.*, 1996; WHO, 2003; Sanders *et al.*, 2016; Wilkinson *et al.*, 2016; Robinson *et al.*, 2019b). Differential, but still constant, discounting has also been recommended, where costs are discounted using a higher discount rate relative to health effects (WHO, 2019), which can be justified on the basis of expected growth in $k$ or $v$ (Claxton *et al.*, 2011). However, recommendations around discount rates are a source of much controversy, with the choice of 3% argued to be inappropriate for fast-growing LMICs in particular and arguably lack coherence with relevant theory and empirics more generally (Haacker *et al.*, 2020).

Taken together, we argue that following existing guidelines for economic evaluation in LMICs will result in sub-optimal decisions. This is particularly important as resources available for healthcare are especially limited in LMICs compared to high-income countries, and projected economic growth can outstrip more mature economies with implications for expected changes in the marginal cost per unit of health produced by the healthcare sector, expected changes in the consumption value of health, and discount rates. What is lacking is 3-fold: clarity regarding the separate role for key parameters $k$ and $v$, guidance as to plausible assumptions about how these parameter values might evolve over time for a given country, and discount rates that reflect these and other country-specific factors.

The paper is structured as follows. The ‘Methods’ section contains two parts. The first part outlines a conceptual economic evaluation framework that aims to clarify the distinct roles of different evaluation parameters in evaluating a project based on Claxton *et al*. (2019). The second part describes a comparison of alternative evaluation approaches (i.e. first, our preferred approach, which uses parameters based on the conceptual economic evaluation framework, and then approaches parameters implied by the existing guidelines both where the objective is health and where it is consumption) applied to a simple hypothetical project. Correspondingly, the ‘Results’ section first provides estimates for each of these parameters [the marginal cost per unit of health produced by the healthcare sector ($k$), the consumption value of health ($v$), the discount rate for health (${r_h}$) and the discount rate for consumption value (${r_c}$)], for each country and in each time period, based on available empirical evidence for both the preferred approach and other approaches inspired by the assumptions from guidelines. Where existing estimates are not available for future values of the marginal cost per unit of health produced by the healthcare sector ($k$), new estimates are obtained following a practical method for obtaining projected values of this parameter for each country over time. The remainder of this paper demonstrates an application of this framework to calculate the estimated net benefit of a hypothetical project across LMICs. This serves to illustrate the sub-optimal consequences of alternative assumptions about key parameters that result from adhering to recommendations from existing guidelines. All parameter values used within this application are provided in the Supplementary material.

## Methods

This section first sets out the economic evaluation framework that identifies key parameters for which country- and time-specific estimates are required and data sources for these are identified. This informs our preferred approach. Second, we describe a method to evaluate existing guidelines in order to expose implicit assumptions about these parameters that are embedded within existing guidance through enabling a like-for-like comparison of the approaches implied within current guidelines with our preferred approach. As part of this, a method is outlined for comparing the results from our preferred assumptions to those in existing guidelines both when the objective is to improve health and when it is to improve consumption value.

### Framework of evaluation

Where the objective is to improve health, evaluation focuses on the health achieved by the project net of health opportunity costs. In a given year, for a given country, this is calculated as follows:

(1) \begin{equation*}NH{B_{i,t}} = {h_{i,t}} - {c_{i,t}}/{k_{i,t}}\end{equation*}

For each country, $i$, in year $t$, ${c_{i,t}}$ is the total additional cost of the project, ${h_{i,t}} $ is the total DALYs averted by the project and ${k_{i,t}}$ is the marginal cost per unit of health produced by the healthcare sector. By specifying all of these parameter values as for a given country and year, it is indicated that each may potentially vary by country as well as over time. In Equation (1), all parameters are undiscounted. From the estimated time profile of net health effects, $NH{B_{i,t}}$, a net present value can be calculated for each country using the discount rate for health (${r_h}_{i,t}$):

(2) \begin{equation*}NHB_i^{NPV} = \mathop \sum \limits_{t = {t_0}}^T {{NH{B_{i,t}}} \over {{{\left( {1 + {r_h}_{i,t}} \right)}^{t - {t_0}}}}}\end{equation*}

${t_0}$ and $T$ represent the first and final years within the time horizon of the economic evaluation, respectively. The discount rate itself is allowed to vary for each country and over time.

Where the objective is to increase consumption, for each time period, for a given country, it is necessary to evaluate the consumption value of the net health benefits of the project:^1^

(3) \begin{equation*}NC{B_{i,t}} = {v_{i,t}}*NH{B_{i,t}}\end{equation*}

 

(4) \begin{equation*}NCB_i^{NPV} = \mathop \sum \limits_{t = {t_0}}^T {{NC{B_{i,t}}} \over {{{\left( {1 + {r_c}_{i,t}} \right)}^{t - {t_0}}}}}\end{equation*}

For each country, $i$, in year $t$, ${v_{i,t}}$ is the consumption value of a DALY. A net present value can be calculated for each country from the time profile of equivalent consumption effects using the discount rate for consumption (${r_c}_{i,t}$).

#### **Marginal cost per unit of health produced by the healthcare sector**, $\ {k_{i,t}}$

We use estimates of cost per DALY averted from 2015 that reflect ${k_{i,2015}}$ based on two similar studies (Ochalek *et al.*, 2018; Ochalek and Lomas, 2020). There is a considerable variation exhibited among LMICs, ranging between $59 and $17 058 per DALY averted (2017 USD) in Guinea-Bissau and Costa Rica, respectively (Ochalek *et al.*, 2018).^2^ However, no published estimates of future values of ${k_{i,t}}$ are available. Changes in ${k_{i,t}}$ over time depend on many factors, which makes future values difficult to anticipate (Paulden *et al.*, 2017). This paper offers a practical method for obtaining future projections of ${k_{i,t}}$ by analysing the relationship^3^ between estimates of ${k_{i,2015}}$ and GDP per capita as well as total fertility rate, for both of which projected values exist (Dieleman *et al.*, 2017), across countries, and sub-groups of countries.^4^ Using this relationship, we are able to project annual estimates of ${k_{i,t}}$ for 97 LMICs from 2015 to 2040. Employing these values avoids relying on simplifying assumptions such as ${k_{i,t}}$ remaining constant over time or growing at a constant rate (see Supplementary Appendix A).

#### **Consumption value of health**, ${v_{i,t}}$

Although not the only way to obtain a consumption value of health, here, we use recently published estimates of the value of a statistical life (VSL) in 2015, which are available for 95 LMICs based on extrapolation of an estimate for USA using gross national income per capita (Robinson *et al.*, 2019a). For application to net health effects denominated in DALYs, it is advised to calculate the value of a statistical life year (VSLY), which can then form the basis of estimates of ${v_{i,t}}$ (Robinson *et al.*, 2019a). Following Robinson *et al*. (2019a), ${v_{i,2015}}$ is calculated by dividing VSL by the conditional life expectancy at the age equal to half of the life expectancy at birth to obtain VSLY.^5^

There is a related literature that analyses the income elasticity of $v$, which can be drawn upon to provide estimates of ${v_{i,t}}$ for 2015–40, given the availability of projected estimates of GDP per capita (Hammitt and Robinson, 2011; Viscusi and Masterman, 2017; Masterman and Viscusi, 2018; Robinson *et al.*, 2019). In this paper, following Claxton *et al*. (2019a), we consider two different values for the income elasticity of $v$: 1 and 1.5 (with our preferred approach using an elasticity of 1). An elasticity of 1 reflects that $v$ increases by the same proportion as GDP per capita, while an elasticity >1 reflects that health is a luxury good, with $v$ increasing by a greater proportion than the increase in GDP per capita.

#### **Discount rate for health**, $\ {r_h}_{i,t}$

Where the objective is to improve health, Paulden and Claxton (2012) argue that net health should be discounted at a rate, ${r_h}_{i,t}$, that reflects the interest rate faced by the payer, ${r_s}_{i,t}$, minus the growth rate of $k$, ${g_k}_{i,t}$:

(5) \begin{equation*}{r_h}_{i,t} \approx {r_s}_{i,t} - {g_k}_{i,t}\end{equation*}

The growth rate of $k$, ${g_k}_{i,t}$, can be derived from estimates of ${k_{i,t}}$. Without estimates for ${r_s}_{i,t}$, we are required to make an assumption to obtain a suitable proxy based on the compound annual growth rate (CAGR) of GDP per capita from the projected estimates of GDP per capita (${g_c}_{i,t}$). The rationale for this is that as ${r_c}_{i,t} = {g_c}_{i,t}$, where $\rho = 0$ and $\eta = 1$, we are effectively proxying ${r_s}_{i,t}$ with ${r_c}_{i,t}$, which is the mirror image of the assumption made by the Council of Economic Advisers in 2017 who proxy ${r_c}_{i,t}$ with ${r_s}_{i,t}$ (Council of Economic Advisers, 2017).

#### **Discount rate for consumption value**, $\ {r_c}_{i,t}$

Where the objective is to improve consumption, the discount rate, ${r_c}_{i,t}$, reflects the social time preference rate for consumption and can be based on the Ramsey Rule that comprises a pure time preference rate, $\rho $, and a wealth effect that is the product of the growth rate of future consumption (${g_c}_{i,t}$) and the weight that ought to be attached to it ($\eta $), $\eta *{g_c}_{i,t}$:

(6) \begin{equation*}{r_c}_{i,t} = \rho + \eta *{g_c}_{i,t}\end{equation*}

There is a consensus that pure time preference rate, $\rho $, is most appropriately considered to be small or zero for social decision-making (Drupp *et al.*, 2018). The wealth effect requires consideration of the appropriate basis and value for $\eta $. In the context of social decision-making, empirical estimates of social inequality aversion have been used to infer values for $\eta $, which suggest $1 \lt \eta \lt 2$ (Groom and Maddison, 2019). According to this basis for ${r_c}_{i,t}$, discount rates should be higher in countries with higher expected economic growth, and therefore, the commonplace usage of 3% per annum may be too low for fast-growing LMICs (Haacker *et al.*, 2020). Our preferred approach employs a value of $\rho $ equal to 0 and $\eta $ equal to 1.

### Evaluating existing guidelines for economic evaluation

To illustrate the importance of different assumptions and estimates when applying this flexible evaluation framework to a project, we compare our preferred approach to other approaches inspired by existing guidelines. The comparison is undertaken for two different types of analysis: one where the objective is to improve health and the other to improve consumption.

#### A simple hypothetical health-related project

For all countries analysed, we assume that a project affects 10% of each country’s population in 2015. The additional costs and DALYs averted per affected individual are $25 (2017 USD) prices and 0.1, respectively, each year for the period 2015–40. It is assumed that the project occurs throughout the 2015–40 period or not at all. This example is highly stylized with its ICER being invariant to the discount rate so long as the same discount rate is used for healthcare costs and health gains ($250 per DALY averted, which is similar to the median value of ${k_{i,2015}}$ among low- and lower-middle-income countries of $307 per DALY averted).

#### Enabling like-for-like comparison across guidelines

When the objective is to improve health, our framework of evaluation requires values for ${k_{i,t}}$ in order to calculate the time profile of net health effects [Equation (1)] for each country and ${r_h}_{i,t}$ to convert this into a net present value [Equation (2)]. When the objective is to improve consumption, our framework instead requires values for both ${k_{i,t}}$ and ${v_{i,t}}$ in order to calculate the time profile of net consumption effects [Equation (3)] and ${r_c}_{i,t}$ to convert this into a net present value [Equation (4)].

This differs from what is conventionally done in economic evaluation, which means that a like-for-like comparison of the estimates of net health and net consumption benefit that result from the application of existing guidelines for economic evaluation in LMICs requires inferring assumptions implied about the growth rates of $k$ or $v$, which can be revealed from the recommended discount rates using Claxton *et al*.’s (2011) framework (although it is important to note that the resulting inferred assumptions do not necessarily reflect the intention of the authors).

Claxton *et al*. (2011) argue that the cost-effectiveness threshold most appropriately represents $k$ when the objective is to improve health, given an exogenous budget constraint. Their framework shows that, in this context, the discount rate for healthcare costs is given by ${r_{hc}} \approx {r_h} + {g_k}$. This implies that a choice of differential discounting in guidelines with a lower discount rate for health indicates an expectation of growth in $k$, while applying the same discount rate to health and healthcare reveals an implicit assumption that $k$ will remain constant in real terms.

Using this method, we were able to parameterize two evaluation approaches with the objective of improving health based on the iDSI (Wilkinson *et al.*, 2016) ‘iDSI_H’ and WHO immunization (WHO, 2019b) ‘WHOi_H’ guidelines to compare against our preferred approach ‘Preferred_H’.

Where the objective is to increase consumption, there can be up to three separate discount rates for consumption (${r_c}$), health gains (${r_h}$), and healthcare costs (${r_{hc}}$), which are characterized in existing guidelines as uniform across countries and constant over time. The discount rate for health gains is found to be ${r_h} = {r_c} - {g_v}$, which indicates that if $v$ is expected to grow, then relatively more weight should be given to future health than should future consumption. If the budget is considered exogenous, the discount rate for healthcare costs is given by ${r_{hc}} \approx {r_h} + {g_k}$, which implies that in this context ${r_{hc}} \approx {r_c} - {g_v} + {g_k}$. Again, a difference between the recommended discount rates for health and healthcare costs indicates an implicit assumption about the growth of $k$. In this context, the assumption regarding the growth rate of $v$ is revealed by differences between the discount rate for consumption and the other recommended discount rates. If the budget is considered endogenous, then the discount rates for healthcare costs and consumption are the same (${r_{hc}} = {r_c}$) and a lower discount rate for health implies an expectation of growth in $v$.

Using this method, we are able to parameterize three evaluation approaches with the objective of improving consumption based on the WHO Generalized CEA (GCEA) (WHO, 2003) ‘WHO_C’, WHO immunization (WHO, 2019) ‘WHOi_C’ and BCA (Robinson *et al.*, 2019b) ‘BCA_C’ guidelines to compare against our preferred approach ‘Preferred_C’.

## Results

This section first presents the results of parameterizing the different evaluation approaches, our preferred approach and the approaches inspired by existing guidelines and then presents the results of an application of each to the economic evaluation of a hypothetical project.

### Parameterizing the evaluation approaches

#### Preferred approach

Estimates for ${g_c}_{i,t}$ are based on Dieleman *et al*. (2017) and enable calculation of values for evaluation parameters ${k_{i,t}}$, ${v_{i,t}}$, ${r_c}_{i,t}$ and ${r_h}_{i,t}$ for 95 LMICs. These results are provided in full in Supplementary Appendix B and are summarized below for two countries chosen for illustrative purposes: Bangladesh and Yemen.

In 2015, both countries are lower-middle-income countries that are eligible for GAVI support. Bangladesh is estimated to have a $k$ in 2015 of $142 per DALY compared to $241 per DALY for Yemen (2017 USD) (Ochalek *et al.*, 2018). The estimated $v$ in 2015 is $1272 for Bangladesh and $1090 for Yemen (2017 USD) (Robinson *et al.*, 2019a).

Analysis of the two countries over time reveals some differences. Both countries are projected to have decreases in total fertility rate with a CAGR of −0.8% in Bangladesh and −1.9% in Yemen. The major difference between these two countries lies in the forecasted trajectories of economic growth where Bangladesh is expected to grow at a CAGR of 4.6% over the period 2015–40, while Yemen is expected to experience a CAGR of −0.2% (Yemen’s annual growth rate is negative until 2020 and positive thereafter).

In our preferred approach with the objective of improving health, ‘Preferred_H’, these projected changes lead to differences in the trajectories of the evaluation parameters $k$ and ${r_h}$ over time. In Yemen, $k$ is projected to initially fall and then rise, with a CAGR over the whole period of 0.7% ($k$ for Yemen in 2040 is projected to be $286 per DALY in 2017 USD). In contrast, in Bangladesh, $k$ is projected to increase throughout with a CAGR of 4.7% ($k$ for Bangladesh in 2040 is projected to be $443 per DALY in 2017 USD). Negative values for ${r_h}$ are estimated for Bangladesh and Yemen throughout, with the magnitude falling between 2016 (−0.6% for Bangladesh and −1.7% for Yemen) and 2040 (−0.1% for Bangladesh and −0.9% for Yemen).

The differences in projected changes over time also impact upon the trajectories of $v$ and ${r_c}$, which are required for our preferred approach with the objective of improving consumption ‘Preferred_C’. With the assumptions of the income elasticity of $v$ and $\eta $ equal to 1, these trajectories exactly mirror that projected for economic growth. This means that $v$ grows with a CAGR of 4.6% in Bangladesh ($v$ for Bangladesh in 2040 is projected to be $3898 in 2017 USD) but falls with a CAGR of −0.2% in Yemen ($v$ for Yemen in 2040 is projected to be $1033 in 2017 USD). Yemen has negative values for ${r_c}$ throughout the period analysed falling in magnitude from −11.7% in 2016 to −0.2% in 2040. In contrast, Bangladesh has positive values throughout falling from 6.1% in 2016 to 4.6% in 2040.

#### Assumptions implicit within existing guidelines

##### ‘iDSI_H’

The results from this section are summarized in Tables 1 and 2 with more detail given below. The iDSI reference case (Wilkinson *et al.*, 2016) recommends that an ICER is calculated using a discount rate of 3% for both healthcare costs and health gains and that this is compared to a cost-effectiveness threshold that reflects $k$. This closely relates to our evaluation framework when the objective is to maximize health. The use of the same discount rate for healthcare costs and health gains therefore implies an assumption of no growth in $k$ (i.e. ${g_k} = 0$ and ${r_h} = 3\% $).

**Table 1.** Approaches to analysis where the objective is to improve health

Analysis	Values for ${{\boldsymbol{{k}}}_{{\boldsymbol{{i}}},{\boldsymbol{{t}}}}}$	Values for ${{\boldsymbol{{r}}}_{\boldsymbol{{h}}}}_{{\boldsymbol{{i}}},{\boldsymbol{{t}}}}$
iDSI_H	${k_{i,2015}}$	3%
WHOi_H	${k_{i,2015}}*{\left( {1.03} \right)^{t - 2015}}$	0%
Preferred_H	${k_{i,t}}$	${r_s}_{i,t} - {g_k}_{i,t}$

**Table 2.** Approaches to analysis where the objective is to improve consumption^a^

Analysis	Values for ${{\boldsymbol{{k}}}_{{\boldsymbol{{i}}},{\boldsymbol{{t}}}}}$	Values for ${{\boldsymbol{{v}}}_{{\boldsymbol{{i}}},{\boldsymbol{{t}}}}}$	Values for ${{\boldsymbol{{r}}}_{\boldsymbol{{c}}}}_{{\boldsymbol{{i}}},{\boldsymbol{{t}}}}$
WHO_C	${k_{i,2015}}$	${v_{i,2015}}$	3%
WHOi_C	${k_{i,2015}}*{\left( {1.03} \right)^{t - 2015}}$	${v_{i,2015}}*{\left( {1.03} \right)^{t - 2015}}$	3%
BCA_C	${k_{i,t}}$	${v_{i,t}}$ (income elasticity = 1.5)	3%
Preferred_C	${k_{i,t}}$	${v_{i,t}}$ (income elasticity = 1)	${g_c}_{i,t}$

a Our preferred approach (‘Preferred_C’) takes assumptions for the income elasticity of $v$ and ${r_{{c_{i,t}}}}$ from the ‘conservative scenario’ in Claxton *et al*. (2019). We present the results of an additional three analyses using different assumptions suggested in Claxton *et al*. (2019) in Supplementary Appendix C.

##### ‘WHO_C’

The WHO GCEA guide (WHO, 2003) recommends a similar approach, with discount rates of 3% for both healthcare costs and health gains, but argues that the objective ought to be consumption and that the budget for health should not be considered exogenous. In this context, the lack of divergence between the discount rates for healthcare costs and health gains implies an assumption of $v$ remaining constant in real terms (i.e. ${g_v} = 0$ and ${r_c} = 3\% $). We construct an analysis where the objective is to improve consumption but allow for consideration of an exogenous budget constraint in order to fit within our framework.

##### ‘WHOi_H’ and ‘WHOi_C’

More recently, WHO has released separate guidelines for evaluating immunizations (WHO, 2019). These guidelines differ in two important ways for our purposes. First, the guidelines refer to the use of a cost-effectiveness threshold but do not state the appropriate basis for this. Second, they propose differential discounting with a discount rate for health gains equal to 0% with discount rates of 3% for healthcare costs and consumption (${r_h} = 0\% $, ${r_{hc}} = 3\% $ and ${r_c} = 3\% $). The use of differential discounting in this way implies an assumption of 3% annual growth in both $k$ and $v$ (i.e. ${g_k} = 3\% $ and ${g_v} = 3\% $) when viewed through the lens of Claxton *et al*.’s (2011) framework.

##### ‘BCA_C’

Finally, the BCA guidelines (Robinson *et al.*, 2019a) differ from other guidelines in that its recommendations are not based on the conventional practice of CEA. Instead, it is recommended that health gains are converted into their consumption value and that $v$ is adjusted for expected growth in GDP per capita using an income elasticity of the consumption value of health of 1.5. No role is acknowledged for an exogenous budget constraint, but this is required for our framework. As such we assume the same principle of projecting $k$ where possible. These guidelines recommend a constant discount rate of 3% for all countries (i.e. ${r_c} = 3\% $).

### Comparing the results from the evaluation approaches when applied to the simple hypothetical health-related project

The proportion of countries where a positive net present value of net health benefits is estimated varies according to the evaluation approach that is taken. This is summarized in Table 3.

**Table 3.** Proportion of countries where a positive net health benefit is estimated by income category by evaluation approach

	Proportion of countries where a positive net health benefit is estimated		
Groups of countries	iDSI_H (%)	WHOi_H (%)	Preferred_H (%)
Low-income	25	42	42
Lower-middle-income	81	92	89
Upper-middle-income	100	100	100
LMICs (all)	74	82	81

Positive net health benefits are generated in all upper-middle-income countries and in most lower-middle-income countries and negative net health benefits in the majority of low-income countries regardless of approach. This is because ${k_{i,t}} $tends to be lower in low-income countries than in lower-middle-income countries, which are in turn lower than in upper-middle-income countries.

The observed differences in the sign of the net health benefit across approaches summarized in Table 3 are driven by the different assumptions about the projected trajectory of $k$ over time. Assuming that $k$ is constant in real terms over time (as in ‘iDSI_H’) generally underestimates its growth compared to using our projected values of ${k_{i,t}} $ (as in ‘Preferred_H’). In contrast, the assumption of 3% annual growth (as in ‘WHOi_H’) may underestimate or overestimate depending on the actual growth rate in each year in each country. Results by country are reported in Supplementary Appendix C, Table C2. Yemen provides an interesting case with the sign of its estimated net health benefit differing between evaluation approaches. In the case of Yemen, the 3% annual growth rate in $k$ represents an overestimate compared to our projected estimates, which forecasts 0.7% CAGR. This has an impact on the estimated sign of the net health benefit, with only the ‘WHOi_H’ approach giving a positive net health benefit.

**Figure 1.**
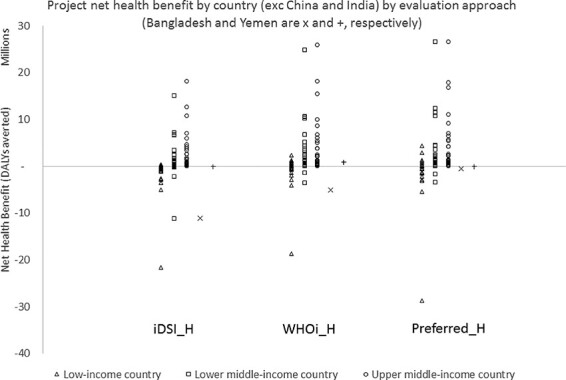
Net health benefit by country by evaluation approach

Differences are also observed between the results of the three approaches for each country when we consider the estimated net health benefit itself (and not just its sign). The results by country in Supplementary Appendix C, Table C2 are summarized in Figure 1.

Moving from considering the sign of net health benefit to its magnitude, the value of the project in terms of the net present value of net health benefit is higher for each country when analysed according to ‘WHOi_H’ compared to ‘iDSI_H’ for two reasons. First, as explained above, assuming that $k$ is constant (as in ‘iDSI_H’) results in lower estimated net health benefits compared to when it is assumed to grow at 3% per year in ‘WHOi_H’. Second, this is compounded by the lower discount rate applied to net health benefit in ‘WHOi_H’, which means that there is a higher weight attached to future values of net health benefit where $k$ is highest. The results for ‘Preferred_H’ are more similar to ‘WHOi_H’, because the assumption of 3% growth in $k$ implied by ‘WHOi_H’ is not far off the unweighted CAGR across countries of 2.3% from our projected estimates. However, this masks considerable variation in the forecasts (range: −0.5% to 6%, see Supplementary Appendix A, Table A3), meaning that for some countries the ‘WHOi_H’ will result in an underestimate of the net present value of net health benefit, while for others, it will result in an overestimate. With respect to the discount rate adopted, the country-specific discounting in ‘Preferred_H’ applies a discount rate to net health close to 0% (0% is also applied in ‘WHOi_H’) for all countries because ${r_s}_{i,t}$ and ${g_k}_{i,t}$ almost exactly offset. This is an artefact of how they are both calculated as a function of projected growth in GDP per capita (see Supplementary Appendix A, careful inspection finds that the country-specific discounting in ‘Preferred_H’ often indicates a small negative discount rate where ${g_k}_{i,t} \gt {r_s}_{i,t}$).

For approaches to evaluation where the objective is to increase consumption, we find a similar pattern in terms of the sign of the estimated net consumption benefit across countries as was found when considering net health benefit. This is summarized in Table 4.

**Table 4.** Proportion of countries where a positive net consumption benefit is estimated by income category by evaluation approach

	Proportion of countries where a positive net consumption benefit is estimated			
Groups of countries	WHO_C (%)	WHOi_C (%)	BCA_C (%)	Preferred_C (%)
Low-income	25	42	42	42
Lower-middle-income	81	92	92	89
Upper-middle-income	100	100	100	100
LMICs (all)	74	82	82	81

**Figure 2.**
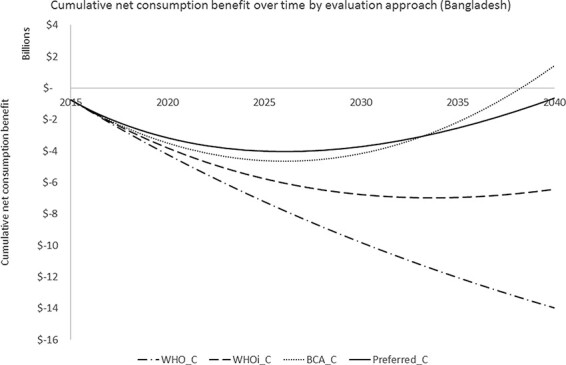
Cumulative net present value of net consumption benefit over time for Bangladesh according to different evaluation approaches

This project generates positive net consumption benefits in the majority of middle-income countries (in all upper-middle-income countries and in most lower-middle-income countries) and negative net consumption benefits in the majority of low-income countries. The reason for this finding is again because of the different values of ${k_{i,t}}$ that are generally found across income categories as it is ${k_{i,t}}$ that determines if (and when) a net health benefit is achieved. With this project, there are no wider effects beyond health and so calculating net consumption benefit effectively only involves re-scaling these net health benefits. The proportion of countries where a positive net health benefit is estimated is therefore lowest according to ‘WHO_C’, which illustrates the effect of assuming no real terms changes in ${k_{i,t}}$. The differences between the results from the other approaches presented here are more subtle. As with the analysis with the objective of improving health, Yemen is only found to have a positive estimated net consumption benefit according to ‘WHOi_C’. Again, this illustrates that the assumed growth rate of 3% for $v$ and $k$ is likely to be an overestimate for some countries such as Yemen. Another interesting case is that a positive net consumption benefit is estimated for Bangladesh with the ‘BCA_C’ approach but not with any of the other approaches presented here. To help to understand this finding, we have presented the cumulative net present value of net consumption benefit over time for Bangladesh in Figure 2.

In the ‘BCA_C’ analysis, country-specific projections of ${k_{i,t}}$ are applied (where it is found to grow at a CAGR of 4.7%), which results in net consumption benefits being generated from 2027 onwards (the turning point for ‘BCA_C’ and also ‘Preferred_C’ in Figure 2). In addition, $v$ is assumed to grow to a disproportionately high extent with GDP growth (with an income elasticity of health of 1.5), where Bangladesh is forecasted to experience a high growth in GDP over the period under consideration (CAGR of 4.6%). Finally, the uniform discount rate of 3% applied in ‘BCA_C’ is relatively low compared to when based on Bangladesh’s high forecasted GDP growth (e.g. ‘Preferred_C’). Taken together, the ‘BCA_C’ analysis results in net health benefits from 2027, which is the same as in ‘Preferred_C’, that are valued more highly and discounted less than in ‘Preferred_C’.

**Figure 3.**
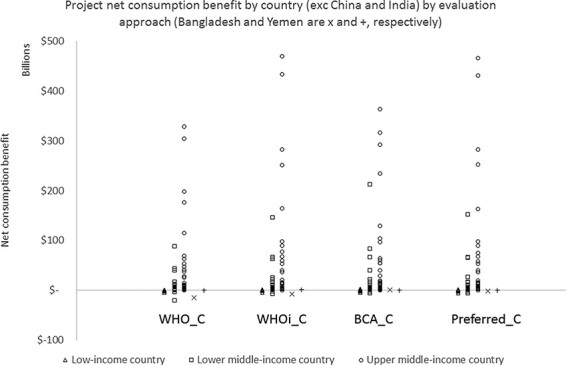
Net consumption benefit by country by evaluation approach

We can also compare the estimated levels of net consumption benefit by country, which is shown in Figure 3.

The discounting strategy is the same for all of ‘WHO_C’, ‘WHOi_C’ and ‘BCA_C’ and so differences in the estimated net consumption benefit are entirely driven by different assumptions about how ${v_{i,t}}$ and ${k_{i,t}}$ might be expected to change over time. As a result, in almost all countries, the magnitude of net consumption benefit is lowest in ‘WHO_C’, which assumes no temporal change in these evaluation parameters. The net consumption benefit appears similar when estimated using ‘WHOi_C’ and ‘BCA_C’, since both involve growing values for ${v_{i,t}}$ and ${k_{i,t}}$ for most countries. The same is also true for ‘Preferred_C’, but a slightly different pattern is observed because the effect of growing values for ${v_{i,t}}$ and ${k_{i,t}}$ is offset by higher discount rates in ‘Preferred_C’ (and conversely slower growth in these values offset by lower discount rates).

## Discussion

Assessing the value of a new health-related project requires information about the project itself, the additional costs it imposes and DALYs averted, but it also requires other information in the form of evaluation parameters. Evaluation parameters are required in order to account for the resource constraints of the healthcare system, preferences of the population served by the decision-maker and the timing of costs and effects. Good examples of conventional practice may use the latest recommendations for evaluation parameters, but they rarely explicitly consider whether these are reasonable, given the country context and the timing of effects of the decision. In particular, conventional practice embeds assumptions about expectations of changes in ${k_{i,t}}$ and ${v_{i,t}}$ over time within discount rates. In part, this has been the approach taken due to the absence of evidence that might inform values for these evaluation parameters for each country over time. However, it has also stifled debate as to whether the implicit assumptions are appropriate for a country at a given point in time, or, indeed, broadly appropriate for LMICs. For example, the widespread use and recommendation of a 3% discount rate are thought to derive from experience with high-income countries and may not be appropriate for LMICs where projected economic growth outstrips more mature economies (Haacker *et al.*, 2020). This paper provides estimates for all of the relevant evaluation parameters for 95 LMICs between 2015 and 2040 and applies them within a formal evaluation framework. A highly stylized hypothetical health-related project is analysed to assess the appropriateness of assumptions implied by recent economic evaluation guidelines.

We reflect conventional practice drawing upon three sources of commonly used guidance: iDSI, WHO and BCA. With the exception of BCA, these approaches adopt uniform assumptions across countries about the growth rate of ${v_{i,t}}$ and ${k_{i,t}}$, and without exception, they advise uniform and constant discount rates (in the absence of existing country guidance).

Empirically derived values for ${k_{i,2015}}$ and ${v_{i,2015}}$ are available, but projected values of ${k_{i,t}}$ and ${v_{i,t}}$ over time have not been available prior to this paper. However, this does not justify assumptions of constancy (or some uniform growth rate across countries) over time when available data can be used to inform estimates of how these might evolve in the future. We apply this principle to estimating ${k_{i,t}}$ in Supplementary Appendix A. We find that allowing ${k_{i,t}}$ and ${v_{i,t}}$ to vary, typically growing, over time shows that the assumption of constancy is likely to underestimate the value of new projects. In analyses devised to inform objectives of improving health and consumption, approaches that allow ${k_{i,t}}$ and ${v_{i,t}}$ to vary with time are found to produce higher estimates of net health and net consumption effects, with the sign changing from negative to positive in 7–8% of countries analysed using our hypothetical project. The hypothetical project analysed is highly stylized and simplistic; it is likely that the effect of different assumptions about evaluation parameters would be greater in the case of projects such as vaccines where results are particularly sensitive to the handling of the timing of costs and effects (WHO, 2019).

The country- and time-specific values we have used for ${k_{i,t}}$, ${v_{i,t}}$, ${r_h}_{i,t}$ and ${r_c}_{i,t}$ are available in Supplementary Appendix B. A number of assumptions pertain to these values with our method for projecting ${k_{i,t}}$ detailed in Supplementary Appendix A. We have also assumed that the projected growth in GDP per capita can be used as a proxy for ${r_s}_{i,t}$ when calculating ${r_h}_{i,t}$. Another assumption concerns the use of the Ramsey Rule to calculate values for ${r_c}_{i,t}$. The Ramsey Rule is well-established in economics, with its basis founded on society’s preferences, but there is no guarantee that these will be reflected in the ability of decision-makers to allocate resources inter-temporally (i.e. it is not guaranteed that ${r_c}_{i,t} = {r_s}_{i,t}$). Other considerations highlighted in Claxton *et al*. (2019) concerning factors to be incorporated in discounting, such as the time horizon under consideration, the relationship between macro-economic risk and project-specific risk and uncertainty around projected growth rates in real income and effects on ${r_c}_{i,t}$, are not considered but are important areas for further research. Parameter uncertainty, more generally, particularly in future values, is not incorporated within this analysis, but it is likely to be considerable and may be of relevance to decision-makers.

## Conclusion

Conventional practice in health economics often embeds implicit assumptions about expected changes in resource constraints and societal preferences within the discount rates used to evaluate projects resulting in a lack of clarity and transparency. In addition, the assumptions made may not be appropriate for a country at a given point in time or for LMICs in general. Separating out these arguments and marshalling available evidence to inform estimates of evaluation parameters by country and over time can produce a flexible evaluation framework that can inform decision-makers in a more transparent and appropriate manner.

This paper provides such a framework and demonstrates (and provides the required parameter values for) the application of this framework. Even when applied to a simple hypothetical example, this framework with preferred assumptions about evaluation parameters gives important differences in results compared to the application of existing guidelines. The implication is that analysts need to consider critically the appropriateness of existing guidelines for economic evaluation in the context of their country. In addition, looking ahead, future guidelines for economic evaluation in LMICs should ensure that assumptions about evaluation parameters are clearly and explicitly stated and are founded on the best available evidence.

## Supplementary Material

czab104_Supp

## Data Availability

The data underlying this article are available in the article and in its online supplementary material.

## Endnotes

1. For simplicity, in this example, there are no non-health effects to consider, but in general, they would be incorporated here (Claxton *et al.*, 2019; Walker *et al.*, 2019).

2.
Woods *et al*. (2016) provide estimates for a range of countries (including LMICs) based on extrapolation of the Claxton, Martin, *et al*. (2015) estimate for the UK. Estimates for some (mainly high-income) countries exist that use bespoke within-country analysis (Claxton *et al.*, 2015; Edney *et al.*, 2018; Vallejo-Torres *et al.*, 2018; Siverskog and Henriksson, 2019; Stadhouders *et al.*, 2019; van Baal *et al.*, 2019; Edoka and Stacey, 2020; Ochalek *et al.*, 2020).

3. We tested for structural breaks in the relationship between $k$ and GDP per capita by categorizing countries according to eligibility for support funding from different institutions: GAVI, IMF and Global Fund. We ultimately derive a satisfactory model that differs for GAVI-eligible countries (see Supplementary Appendix A). These countries are those that have access to GAVI-negotiated prices for vaccines.

4. The compound annual growth rate of GDP per capita between 2015 and 2040 varies considerably among LMICs, ranging between −0.5% (Congo) and 5.3% (China).

5. This is justified as a pragmatic proxy for what is typically done to calculate VSLY from VSL, which is to divide the estimated VSL by the conditional life expectancy at the average individual included in the study.
