# Supplementary material for: Accounting for country- and time-specific values in the economic evaluation of health-related projects relevant to low- and middle-income countries
Source: Health Policy Plan. 2021 Aug 19;37(1):45–54. doi: 10.1093/heapol/czab104 (PMC8757497; doi:10.1093/heapol/czab104)
Supplement: czab104_Supp [file czab104_supp.zip › 210608_projAppC.docx]

## Appendix C – net benefit and global value results in full by evaluation approach

Table C1 – Approaches to analysis where the objective is to improve health

| **Analysis** | **Values for** $\boldsymbol{k}_{\boldsymbol{i,t}}$ | **Values for** ${\boldsymbol{r}_{\boldsymbol{h}}}_{\boldsymbol{i,t}}$ |
| --- | --- | --- |
| iDSI_H | $k_{i,2015}$ | 3% |
| WHOi_H | $k_{i,2015}*{(1.03)}^{t-2015}$ | 0% |
| Preferred_H | $k_{i,t}$ | ${r_{s}}_{i,t}-{g_{k}}_{i,t}$ |
| Intermediate_H | $k_{i,t}$ | 3% |

Table C2 – Net health benefit by evaluation approach by country

|  | **iDSI_H** | **WHOi_H** | **Preferred_H** | **Intermediate_H** |
| --- | --- | --- | --- | --- |
| **Low-income countries** |  |  |  |  |
| Benin | -224,607 | 176,584 | 97,694 | 25,520 |
| Burkina Faso | -1,003,448 | -316,435 | -114,214 | -191,922 |
| Burundi | -1,104,021 | -718,607 | -1,540,167 | -1,007,441 |
| Chad | -997,871 | -462,582 | -1,422,300 | -937,830 |
| Comoros | 6,760 | 36,251 | 19,755 | 11,951 |
| Democratic Republic of the Congo | -21,635,089 | -18,742,809 | -28,800,758 | -18,710,167 |
| Ethiopia | -2,576,582 | 1,248,537 | 4,316,441 | 2,314,578 |
| Guinea | -1,019,652 | -585,984 | -635,621 | -493,910 |
| Guinea-Bissau | -512,714 | -446,814 | -543,536 | -376,276 |
| Haiti | -476,398 | -70,670 | -493,794 | -340,450 |
| Madagascar | -4,948,506 | -4,029,204 | -5,370,885 | -3,750,699 |
| Malawi | -1,164,630 | -529,410 | -1,249,370 | -856,210 |
| Mali | -3,493,228 | -2,831,701 | -3,163,594 | -2,271,822 |
| Mozambique | -280,692 | 745,127 | 673,549 | 354,522 |
| Nepal | 90,701 | 1,115,496 | 1,236,685 | 756,294 |
| Niger | -2,756,465 | -1,997,717 | -2,868,136 | -2,024,229 |
| Rwanda | 6,111 | 437,388 | 608,649 | 360,957 |
| Senegal | 372,331 | 925,354 | 975,410 | 618,196 |
| Sierra Leone | -729,612 | -457,551 | -635,414 | -433,678 |
| Tanzania | 361,034 | 2,313,989 | 2,829,305 | 1,742,274 |
| The Gambia | 27,955 | 107,081 | 75,214 | 45,003 |
| Togo | -559,612 | -281,813 | -404,619 | -306,012 |
| Uganda | -2,909,086 | -1,459,032 | -2,231,504 | -1,646,561 |
| Zimbabwe | -9,803 | 514,245 | 145,770 | 78,564 |
| **Lower middle-income countries** |  |  |  |  |
| Armenia | 215,383 | 326,362 | 317,477 | 224,859 |
| Bangladesh | -10,986,393 | -5,058,926 | -469,619 | -1,433,204 |
| Bolivia | 906,084 | 1,318,419 | 1,381,099 | 929,435 |
| Cambodia | -24,467 | 564,327 | 774,502 | 469,698 |
| Cameroon | -2,223,633 | -1,339,827 | -1,608,205 | -1,228,873 |
| Cape Verde | 43,454 | 63,360 | 70,343 | 44,648 |
| Congo | 370,791 | 555,003 | 574,090 | 371,311 |
| Cote d'Ivoire | -135,223 | 745,843 | 766,894 | 450,175 |
| Egypt | 6,802,750 | 10,309,490 | 11,491,505 | 7,247,485 |
| El Salvador | 539,643 | 779,582 | 848,598 | 548,997 |
| Ghana | 1,111,826 | 2,168,265 | 2,323,961 | 1,512,213 |
| Guatemala | 1,252,614 | 1,869,138 | 2,055,512 | 1,299,617 |
| Honduras | 744,040 | 1,089,732 | 1,145,945 | 763,152 |
| India | 26,667,753 | 76,367,417 | 96,199,241 | 62,553,064 |
| Indonesia | 15,123,959 | 24,925,537 | 26,644,881 | 18,076,368 |
| Kenya | 2,527,758 | 4,343,987 | 4,518,461 | 2,944,925 |
| Kyrgyzstan | 385,602 | 611,573 | 612,402 | 405,857 |
| Lesotho | 118,229 | 196,336 | 208,297 | 138,228 |
| Mauritania | 82,328 | 235,822 | 194,004 | 122,264 |
| Moldova | 229,712 | 337,238 | 313,390 | 233,176 |
| Mongolia | 237,411 | 351,155 | 363,161 | 244,697 |
| Morocco | 2,522,029 | 3,836,967 | 4,309,292 | 2,709,124 |
| Nicaragua | 517,782 | 753,856 | 785,644 | 530,102 |
| Nigeria | -46,248 | 6,825,067 | 1,921,805 | 993,599 |
| Pakistan | -11,127,942 | -3,562,827 | -3,453,744 | -3,402,109 |
| Philippines | 6,644,774 | 10,518,364 | 12,393,107 | 7,635,657 |
| Sri Lanka | 1,644,237 | 2,439,719 | 2,502,050 | 1,737,122 |
| Sudan | 1,066,956 | 2,542,711 | 2,427,583 | 1,547,366 |
| Swaziland | 88,239 | 130,120 | 140,307 | 89,849 |
| Tajikistan | 287,266 | 607,963 | 573,750 | 372,608 |
| Tunisia | 959,059 | 1,383,162 | 1,455,383 | 967,776 |
| Ukraine | 3,406,346 | 5,119,231 | 4,561,680 | 3,378,125 |
| Uzbekistan | 2,305,264 | 3,492,565 | 3,617,652 | 2,458,344 |
| Vietnam | 7,191,587 | 10,707,223 | 10,832,821 | 7,668,386 |
| Yemen | -93,335 | 911,843 | -104,772 | -129,242 |
| Zambia | 746,887 | 1,349,259 | 1,313,403 | 858,001 |
| **Upper middle-income countries** |  |  |  |  |
| Albania | 241,919 | 351,196 | 379,450 | 248,831 |
| Algeria | 3,480,759 | 4,987,812 | 5,465,394 | 3,507,523 |
| Argentina | 3,817,848 | 5,454,028 | 5,747,760 | 3,835,316 |
| Azerbaijan | 756,962 | 1,123,003 | 1,122,192 | 773,121 |
| Belarus | 820,415 | 1,180,397 | 1,159,583 | 824,624 |
| Belize | 31,143 | 44,835 | 47,974 | 31,351 |
| Botswana | 177,232 | 257,680 | 290,325 | 182,384 |
| Brazil | 18,235,473 | 25,991,959 | 26,661,096 | 18,265,323 |
| Bulgaria | 629,815 | 902,107 | 933,674 | 637,427 |
| China | 119,719,511 | 171,735,729 | 192,914,001 | 122,643,738 |
| Colombia | 4,258,533 | 6,061,195 | 6,505,313 | 4,280,741 |
| Costa Rica | 439,776 | 623,673 | 675,310 | 441,488 |
| Dominican Republic | 881,038 | 1,271,066 | 1,444,965 | 903,705 |
| Ecuador | 1,428,615 | 2,043,606 | 2,178,755 | 1,434,621 |
| Gabon | 163,334 | 237,218 | 257,769 | 165,229 |
| Georgia | 253,647 | 394,963 | 387,021 | 270,237 |
| Guyana | 61,834 | 90,946 | 94,055 | 64,064 |
| Jamaica | 241,490 | 351,159 | 362,508 | 243,653 |
| Jordan | 820,486 | 1,172,007 | 1,305,632 | 826,434 |
| Kazakhstan | 1,533,258 | 2,198,731 | 2,390,779 | 1,550,417 |
| Lebanon | 577,874 | 825,686 | 843,500 | 580,519 |
| Macedonia | 181,327 | 260,205 | 267,482 | 183,399 |
| Malaysia | 2,672,075 | 3,820,381 | 4,201,052 | 2,704,121 |
| Mauritius | 109,973 | 157,869 | 168,395 | 111,878 |
| Mexico | 10,842,297 | 15,464,900 | 16,829,873 | 10,903,463 |
| Namibia | 199,970 | 287,785 | 314,938 | 201,918 |
| Panama | 359,010 | 509,551 | 572,841 | 361,221 |
| Paraguay | 582,824 | 836,557 | 932,318 | 592,378 |
| Peru | 2,666,126 | 3,822,011 | 4,238,683 | 2,702,132 |
| Romania | 1,759,852 | 2,511,542 | 2,663,462 | 1,779,628 |
| Russia | 12,724,313 | 18,190,521 | 17,925,944 | 12,755,473 |
| South Africa | 4,685,994 | 6,787,035 | 7,091,049 | 4,711,108 |
| Thailand | 6,037,805 | 8,644,440 | 9,209,242 | 6,116,155 |
| Turkey | 7,045,376 | 10,024,331 | 11,120,195 | 7,103,077 |
| Turkmenistan | 449,407 | 660,522 | 761,391 | 472,052 |

Table C3 - Proportion of countries where a positive net health benefit is estimated by income category by evaluation approach

|  | **Proportion of countries where a positive net health benefit is estimated** | | | |
| --- | --- | --- | --- | --- |
| **Groups of countries** | **iDSI_H** | **WHOi_H** | **Preferred_H** | **Intermediate_H** |
| Low-income | 25% | 42% | 42% | 42% |
| Lower middle-income | 81% | 92% | 89% | 89% |
| Upper middle-income | 100% | 100% | 100% | 100% |
| LMICs (all) | 74% | 82% | 81% | 81% |

Table C4 – Global health value by income category by evaluation approach

|  | **Global health value by income category by evaluation approach** | | | |
| --- | --- | --- | --- | --- |
| **Groups of countries** | **iDSI_H** | **WHOi_H** | **Preferred_H** | **Intermediate_H** |
| Low-income | 864,892 | 7,620,052 | 10,978,472 | 6,307,860 |
| Lower middle-income | 84,739,764 | 177,776,635 | 197,638,240 | 129,526,228 |
| Upper middle-income | 208,887,312 | 299,276,645 | 327,463,923 | 212,408,749 |
| LMICs (all) | 294,491,967 | 484,673,332 | 536,080,635 | 348,242,838 |

Table C5 – Approaches to analysis where the objective is to improve consumption

| **Analysis** | **Values for** $\boldsymbol{k}_{\boldsymbol{i,t}}$ | **Values for** $\boldsymbol{v}_{\boldsymbol{i,t}}$ | **Values for** ${\boldsymbol{r}_{\boldsymbol{c}}}_{\boldsymbol{i,t}}$ |
| --- | --- | --- | --- |
| WHO_C | $k_{i,2015}$ | $v_{i,2015}$ | 3% |
| WHOi_C | $k_{i,2015}*{(1.03)}^{t-2015}$ | $v_{i,2015}*{(1.03)}^{t-2015}$ | 3% |
| BCA_C | $k_{i,t}$ | $v_{i,t}$ (income elasticity = 1.5) | 3% |
| Preferred_C | $k_{i,t}$ | $v_{i,t}$ (income elasticity = 1) | ${g_{c}}_{i,t}$ |
| Preferred_alt1_C | $k_{i,t}$ | $v_{i,t}$ (income elasticity = 1) | ${2*g_{c}}_{i,t}$ |
| Preferred_alt2_C | $k_{i,t}$ | $v_{i,t}$ (income elasticity = 1.5) | ${g_{c}}_{i,t}$ |
| Preferred_alt3_C | $k_{i,t}$ | $v_{i,t}$ (income elasticity = 1.5) | ${2*g_{c}}_{i,t}$ |

Table C6 - Net consumption benefit by evaluation approach by country

|  | **WHO_C** | **WHOi_C** | **BCA_C** | **Preferred_C** | **Preferred_alt1_C** | **Preferred_alt2_C** | **Preferred_alt3_C** |
| --- | --- | --- | --- | --- | --- | --- | --- |
| **Low-income countries** |  |  |  |  |  |  |  |
| Benin | $-134,283,369 | $105,572,550 | $46,348,951 | $51,224,759 | $25,646,405 | $64,212,182 | $34,324,510 |
| Burkina Faso | $-383,541,761 | $-120,949,050 | $-20,174,328 | $-46,355,632 | $-76,563,846 | $-30,935,749 | $-71,417,042 |
| Burundi | $-189,846,086 | $-123,570,873 | $-163,703,810 | $-240,234,737 | $-250,270,568 | $-235,250,401 | $-245,026,038 |
| Chad | $-599,476,872 | $-277,898,671 | $-500,711,766 | $-766,278,173 | $-827,857,319 | $-736,180,278 | $-795,045,099 |
| Comoros | $2,978,861 | $15,975,245 | $5,293,853 | $7,836,641 | $7,809,134 | $7,850,400 | $7,822,834 |
| Democratic Republic of the Congo | $-4,825,953,325 | $-4,180,797,184 | $-4,247,151,012 | $-5,766,295,242 | $-5,687,467,765 | $-5,805,764,774 | $-5,726,055,979 |
| Ethiopia | $-1,073,206,249 | $520,044,582 | $3,190,107,823 | $1,675,794,949 | $690,202,510 | $2,183,612,712 | $905,289,886 |
| Guinea | $-533,901,881 | $-306,828,302 | $-326,867,131 | $-317,471,637 | $-269,562,393 | $-341,933,824 | $-288,666,539 |
| Guinea-Bissau | $-210,122,670 | $-183,115,436 | $-193,208,143 | $-208,216,482 | $-176,594,382 | $-224,254,841 | $-189,363,327 |
| Haiti | $-283,013,732 | $-41,983,158 | $-208,670,967 | $-271,808,820 | $-265,391,337 | $-275,024,787 | $-268,500,312 |
| Madagascar | $-1,239,076,279 | $-1,008,888,500 | $-1,116,653,162 | $-1,267,906,667 | $-1,115,081,907 | $-1,345,169,951 | $-1,178,670,265 |
| Malawi | $-241,469,949 | $-109,765,757 | $-188,418,062 | $-239,148,054 | $-228,540,669 | $-244,474,222 | $-233,530,765 |
| Mali | $-1,870,909,249 | $-1,516,607,381 | $-1,588,428,435 | $-1,592,227,760 | $-1,318,062,463 | $-1,731,745,414 | $-1,421,278,476 |
| Mozambique | $-88,791,510 | $235,706,809 | $194,994,489 | $196,338,445 | $132,499,050 | $228,808,410 | $154,504,909 |
| Nepal | $60,976,276 | $749,924,155 | $909,757,140 | $793,050,844 | $523,346,335 | $930,875,236 | $609,365,212 |
| Niger | $-500,341,157 | $-362,616,568 | $-432,637,229 | $-493,208,838 | $-436,923,641 | $-521,654,437 | $-460,817,012 |
| Rwanda | $2,863,335 | $204,941,298 | $383,639,100 | $268,062,739 | $151,092,161 | $328,108,768 | $182,066,476 |
| Senegal | $266,749,671 | $662,952,595 | $706,941,377 | $658,841,240 | $473,904,861 | $753,144,002 | $535,032,331 |
| Sierra Leone | $-250,768,792 | $-157,260,954 | $-183,179,477 | $-199,392,691 | $-172,224,324 | $-213,199,934 | $-183,837,921 |
| Tanzania | $269,705,970 | $1,728,638,044 | $2,609,266,419 | $2,014,562,939 | $1,234,080,327 | $2,414,617,965 | $1,459,188,975 |
| The Gambia | $8,761,693 | $33,561,107 | $14,836,485 | $21,043,550 | $20,250,149 | $21,441,371 | $20,627,434 |
| Togo | $-166,907,277 | $-84,052,330 | $-110,086,224 | $-114,943,888 | $-100,436,097 | $-122,308,871 | $-106,384,856 |
| Uganda | $-1,347,690,934 | $-675,925,032 | $-901,551,181 | $-970,984,266 | $-859,284,056 | $-1,027,576,345 | $-905,823,381 |
| Zimbabwe | $-7,700,496 | $403,956,511 | $59,806,082 | $100,306,435 | $102,488,091 | $99,217,027 | $101,373,980 |
| **Lower middle-income countries** |  |  |  |  |  |  |  |
| Armenia | $1,281,745,196 | $1,942,179,696 | $1,833,600,498 | $1,900,551,030 | $1,513,880,399 | $2,097,014,800 | $1,657,930,942 |
| Bangladesh | $-13,970,488,557 | $-6,433,018,977 | $1,436,578,841 | $-631,349,800 | $-2,062,936,718 | $98,908,407 | $-1,872,201,752 |
| Bolivia | $3,730,785,075 | $5,428,568,289 | $5,650,975,476 | $5,420,580,979 | $4,104,793,531 | $6,091,331,758 | $4,567,859,837 |
| Cambodia | $-27,590,932 | $636,368,677 | $1,254,842,259 | $845,580,968 | $461,690,119 | $1,042,777,786 | $560,704,466 |
| Cameroon | $-3,121,509,837 | $-1,880,832,835 | $-2,128,778,364 | $-2,156,980,586 | $-1,860,222,906 | $-2,307,911,111 | $-1,976,709,743 |
| Cape Verde | $155,558,515 | $226,817,647 | $216,559,999 | $226,347,136 | $181,587,715 | $249,090,509 | $198,804,708 |
| Congo | $872,568,008 | $1,306,067,852 | $786,036,667 | $1,235,408,498 | $1,332,154,162 | $1,187,558,474 | $1,279,971,995 |
| Cote d'Ivoire | $-193,262,174 | $1,065,966,741 | $1,058,890,071 | $1,028,248,065 | $718,938,322 | $1,185,848,517 | $826,765,749 |
| Egypt | $44,238,975,172 | $67,043,659,701 | $66,384,237,229 | $67,018,413,739 | $52,345,522,088 | $74,484,257,929 | $57,780,944,164 |
| El Salvador | $3,214,905,599 | $4,644,333,943 | $4,304,263,327 | $4,627,740,855 | $3,788,741,362 | $5,053,401,160 | $4,116,082,546 |
| Ghana | $2,162,190,194 | $4,216,667,597 | $4,926,305,600 | $4,300,261,589 | $3,001,181,457 | $4,964,174,257 | $3,411,839,123 |
| Guatemala | $6,851,645,540 | $10,223,955,528 | $8,656,248,588 | $10,082,729,115 | $8,717,961,928 | $10,772,490,076 | $9,285,917,932 |
| Honduras | $1,782,617,794 | $2,610,847,921 | $2,412,763,597 | $2,589,867,119 | $2,116,522,122 | $2,830,019,789 | $2,299,709,312 |
| India | $53,117,957,563 | $152,111,848,563 | $309,982,685,173 | $187,812,865,493 | $101,111,553,437 | $232,521,143,419 | $122,131,973,031 |
| Indonesia | $88,613,215,793 | $146,041,917,555 | $212,696,520,033 | $152,557,392,761 | $95,123,171,893 | $182,097,809,200 | $110,967,715,751 |
| Kenya | $3,221,333,821 | $5,535,907,479 | $5,183,821,309 | $5,389,967,222 | $4,266,316,021 | $5,960,931,258 | $4,689,142,595 |
| Kyrgyzstan | $417,854,010 | $662,724,665 | $499,567,244 | $624,830,394 | $568,344,419 | $653,279,548 | $593,504,864 |
| Lesotho | $169,740,076 | $281,878,355 | $323,711,804 | $285,187,786 | $202,114,287 | $327,661,586 | $229,182,411 |
| Mauritania | $96,982,340 | $277,796,508 | $167,412,780 | $211,465,418 | $189,549,668 | $222,510,315 | $199,237,845 |
| Moldova | $583,433,476 | $856,532,107 | $756,857,397 | $837,812,933 | $700,148,320 | $907,565,464 | $755,120,810 |
| Mongolia | $1,511,900,164 | $2,236,250,702 | $2,045,306,848 | $2,209,107,911 | $1,812,824,606 | $2,410,155,377 | $1,966,281,508 |
| Morocco | $10,337,574,469 | $15,727,390,303 | $16,704,020,874 | $15,821,572,744 | $11,855,406,628 | $17,843,788,882 | $13,212,026,837 |
| Nicaragua | $1,197,599,445 | $1,743,624,914 | $1,721,757,519 | $1,736,247,726 | $1,359,785,415 | $1,927,785,583 | $1,498,200,006 |
| Nigeria | $-151,788,578 | $22,400,332,527 | $3,261,169,393 | $5,543,803,040 | $5,553,635,195 | $5,538,707,982 | $5,548,704,119 |
| Pakistan | $-20,016,250,364 | $-6,408,591,934 | $-5,951,258,733 | $-6,088,845,637 | $-6,224,440,367 | $-6,020,928,775 | $-6,313,366,925 |
| Philippines | $39,532,337,472 | $62,577,821,196 | $83,010,810,093 | $65,097,647,871 | $42,949,621,704 | $76,465,498,032 | $49,548,333,936 |
| Sri Lanka | $10,644,136,657 | $15,793,766,517 | $21,436,923,290 | $15,986,553,618 | $10,332,246,143 | $18,892,487,176 | $11,940,970,121 |
| Sudan | $2,696,068,822 | $6,425,121,699 | $5,100,424,100 | $5,720,229,719 | $4,716,419,043 | $6,228,809,432 | $5,119,694,324 |
| Swaziland | $490,464,917 | $723,256,584 | $540,070,046 | $707,346,786 | $665,076,790 | $728,619,604 | $684,053,019 |
| Tajikistan | $314,400,171 | $665,389,963 | $515,565,566 | $591,356,396 | $498,168,174 | $638,516,749 | $536,321,560 |
| Tunisia | $5,792,420,990 | $8,353,874,471 | $6,603,286,930 | $8,262,438,145 | $7,546,039,377 | $8,623,166,885 | $7,867,043,418 |
| Ukraine | $11,853,966,459 | $17,814,746,432 | $11,802,492,243 | $16,561,095,965 | $16,610,259,042 | $16,537,198,699 | $16,580,941,038 |
| Uzbekistan | $7,320,363,538 | $11,090,634,841 | $12,349,582,254 | $11,108,078,051 | $8,039,295,329 | $12,676,199,741 | $9,060,628,712 |
| Vietnam | $17,559,076,463 | $26,142,902,665 | $40,337,417,575 | $26,643,319,341 | $16,040,077,128 | $32,110,129,980 | $18,802,303,058 |
| Yemen | $-101,756,435 | $994,119,168 | $-99,108,611 | $-120,794,735 | $-159,876,789 | $-102,513,278 | $-137,045,132 |
| Zambia | $1,066,070,178 | $1,925,866,724 | $1,487,166,425 | $1,760,082,500 | $1,524,633,525 | $1,879,019,903 | $1,622,623,900 |
| **Upper middle-income countries** |  |  |  |  |  |  |  |
| Albania | $1,492,811,614 | $2,167,130,299 | $2,471,245,384 | $2,175,301,480 | $1,556,138,924 | $2,492,016,695 | $1,760,047,923 |
| Algeria | $25,522,814,118 | $36,573,346,341 | $29,191,292,499 | $36,350,867,165 | $33,059,288,451 | $38,008,690,115 | $34,515,657,046 |
| Argentina | $115,275,290,873 | $164,677,733,777 | $129,689,631,200 | $163,631,640,962 | $150,219,683,671 | $170,388,227,756 | $156,183,494,066 |
| Azerbaijan | $9,145,985,839 | $13,568,666,487 | $11,069,075,240 | $13,224,907,701 | $11,670,480,109 | $14,009,880,074 | $12,337,476,271 |
| Belarus | $10,640,167,674 | $15,308,859,080 | $12,178,766,685 | $15,108,658,176 | $13,748,370,653 | $15,794,296,976 | $14,359,770,324 |
| Belize | $212,493,542 | $305,913,689 | $228,152,074 | $302,423,957 | $287,165,733 | $310,101,971 | $294,073,709 |
| Botswana | $2,547,397,374 | $3,703,691,426 | $3,849,429,117 | $3,712,917,088 | $2,819,319,334 | $4,168,576,227 | $3,140,369,250 |
| Brazil | $304,582,664,112 | $434,137,352,910 | $316,809,910,152 | $430,935,568,591 | $417,550,780,786 | $437,676,628,825 | $423,805,570,913 |
| Bulgaria | $9,666,028,946 | $13,845,001,949 | $14,755,869,064 | $13,835,754,260 | $10,341,996,679 | $15,618,479,305 | $11,545,369,890 |
| China | $1,840,090,008,923 | $2,639,579,773,029 | $4,610,170,226,705 | $2,668,543,002,742 | $1,488,719,658,156 | $3,279,365,586,895 | $1,771,407,479,508 |
| Colombia | $47,628,872,016 | $67,790,444,724 | $60,469,727,418 | $67,646,410,317 | $57,018,632,700 | $73,028,588,279 | $61,316,253,301 |
| Costa Rica | $9,841,447,757 | $13,956,776,954 | $14,503,495,810 | $13,957,355,540 | $10,618,978,370 | $15,658,825,187 | $11,792,771,510 |
| Dominican Republic | $11,021,170,235 | $15,900,145,542 | $19,616,988,984 | $16,009,705,540 | $10,948,656,567 | $18,604,532,023 | $12,503,829,205 |
| Ecuador | $14,144,203,101 | $20,233,009,111 | $15,140,700,624 | $20,072,109,820 | $19,073,068,575 | $20,575,026,056 | $19,526,838,566 |
| Gabon | $2,403,146,965 | $3,490,208,667 | $2,598,677,786 | $3,437,657,088 | $3,269,099,886 | $3,522,270,000 | $3,348,238,803 |
| Georgia | $1,586,251,341 | $2,470,006,321 | $2,329,735,448 | $2,405,981,998 | $1,907,871,116 | $2,659,127,830 | $2,093,816,216 |
| Guyana | $363,510,479 | $534,653,336 | $574,147,222 | $534,082,898 | $395,802,466 | $604,664,742 | $443,369,571 |
| Jamaica | $1,730,467,448 | $2,516,330,183 | $1,876,650,989 | $2,468,061,600 | $2,337,790,705 | $2,533,486,887 | $2,398,647,725 |
| Jordan | $5,278,207,347 | $7,539,547,757 | $6,318,402,405 | $7,514,093,442 | $6,611,875,809 | $7,969,705,002 | $6,993,602,475 |
| Kazakhstan | $41,080,094,059 | $58,909,913,777 | $54,050,855,919 | $58,742,490,728 | $48,480,604,674 | $63,945,682,533 | $52,475,776,006 |
| Lebanon | $9,085,577,426 | $12,981,788,231 | $10,524,851,416 | $12,893,503,949 | $11,627,399,969 | $13,532,102,539 | $12,190,746,959 |
| Macedonia | $1,580,578,977 | $2,268,133,545 | $2,212,172,184 | $2,260,772,574 | $1,788,580,079 | $2,500,852,310 | $1,963,833,851 |
| Malaysia | $68,695,410,440 | $98,216,813,667 | $103,635,320,158 | $98,298,118,269 | $73,914,367,246 | $110,736,862,174 | $82,489,468,743 |
| Mauritius | $2,365,428,691 | $3,395,631,135 | $4,033,101,562 | $3,405,172,003 | $2,380,079,771 | $3,930,103,955 | $2,703,067,108 |
| Mexico | $198,671,275,202 | $283,374,583,450 | $234,575,291,423 | $282,317,092,871 | $250,505,487,074 | $298,367,254,039 | $264,128,143,726 |
| Namibia | $1,689,667,927 | $2,431,661,794 | $1,928,792,106 | $2,412,180,212 | $2,200,412,484 | $2,518,840,365 | $2,294,376,260 |
| Panama | $9,359,638,027 | $13,284,365,618 | $16,550,647,136 | $13,307,241,510 | $9,031,414,508 | $15,500,494,168 | $10,328,835,505 |
| Paraguay | $3,473,705,460 | $4,985,983,949 | $5,368,825,906 | $4,995,264,569 | $3,712,131,119 | $5,650,109,294 | $4,151,052,349 |
| Peru | $27,694,966,927 | $39,701,973,321 | $39,790,707,924 | $39,700,985,856 | $30,910,683,146 | $44,174,470,581 | $34,094,121,725 |
| Romania | $37,295,024,822 | $53,224,962,236 | $64,049,241,185 | $53,327,468,235 | $36,947,850,534 | $61,721,668,583 | $42,080,170,327 |
| Russia | $328,977,987,867 | $470,302,887,259 | $363,201,897,281 | $465,815,280,488 | $433,514,991,880 | $482,057,324,186 | $448,335,578,063 |
| South Africa | $53,250,355,888 | $77,126,013,292 | $54,282,421,479 | $75,655,648,875 | $74,785,999,440 | $76,091,471,885 | $75,211,347,941 |
| Thailand | $62,814,210,219 | $89,932,287,568 | $96,334,728,433 | $89,973,125,732 | $66,928,270,980 | $101,738,062,665 | $74,966,681,655 |
| Turkey | $177,107,167,474 | $251,992,359,420 | $292,654,797,736 | $252,393,278,625 | $179,257,724,577 | $289,810,337,819 | $202,484,564,843 |
| Turkmenistan | $6,095,821,751 | $8,959,417,135 | $11,600,241,838 | $9,092,881,358 | $6,059,295,426 | $10,649,730,629 | $6,956,996,325 |

Table C7 - Proportion of countries where a positive net health benefit is estimated by income category by evaluation approach

|  | **Proportion of countries where a positive net consumption benefit is estimated** | | | | | | |
| --- | --- | --- | --- | --- | --- | --- | --- |
| **Groups of countries** | **WHO_C** | **WHOi_C** | **BCA_C** | **Preferred_C** | **Preferred_alt1_C** | **Preferred_alt2_C** | **Preferred_alt3_C** |
| Low-income | 25% | 42% | 42% | 42% | 42% | 42% | 42% |
| Lower middle-income | 81% | 92% | 92% | 89% | 89% | 92% | 89% |
| Upper middle-income | 100% | 100% | 100% | 100% | 100% | 100% | 100% |
| LMICs (all) | 74% | 82% | 82% | 81% | 81% | 82% | 81% |

Table C8 – Global health value by income category by evaluation approach

|  | **Global consumption value by income category by evaluation approach** | | | | | | |
| --- | --- | --- | --- | --- | --- | --- | --- |
| **Groups of countries** | **WHO_C** | **WHOi_C** | **BCA_C** | **Preferred_C** | **Preferred_alt1_C** | **Preferred_alt2_C** | **Preferred_alt3_C** |
| Low-income | $612,035,806 | $4,661,272,896 | $8,120,991,718 | $5,787,062,541 | $3,361,319,024 | $7,031,888,072 | $4,009,596,547 |
| Lower middle-income | $320,827,887,915 | $599,729,137,529 | $835,447,871,050 | $624,744,130,912 | $409,947,659,349 | $735,247,858,278 | $469,540,533,636 |
| Upper middle-income | $3,442,409,850,863 | $4,929,387,366,981 | $6,608,636,018,492 | $4,946,457,006,218 | $3,474,199,951,595 | $5,705,914,078,571 | $3,866,621,437,661 |
| LMICs (all) | $3,763,849,774,584 | $5,533,777,777,407 | $7,452,204,881,260 | $5,576,988,199,671 | $3,887,508,929,968 | $6,448,193,824,922 | $4,340,171,567,844 |
