# Supplementary material for: Accounting for country- and time-specific values in the economic evaluation of health-related projects relevant to low- and middle-income countries
Source: Health Policy Plan. 2021 Aug 19;37(1):45–54. doi: 10.1093/heapol/czab104 (PMC8757497; doi:10.1093/heapol/czab104)
Supplement: czab104_Supp [file czab104_supp.zip › 210806_projAppA.docx]

## Appendix A – projecting changes in marginal productivity of public health care expenditure

## Data and methods

Unlike when thinking about $v_{i,t}$, there are no standard assumptions that can be invoked for projecting future values of $k_{i,t}$. Forecasting changes in marginal productivity of public health care expenditure is complicated by the multiple moving parts that are not easily predicted (Paulden et al., 2017). One approach to forecasting could be informed by the evolution over time of existing empirical estimates of this kind for a given country, but such a time series is only presently available for the English NHS with no clear pattern over time observed (Lomas, Martin, & Claxton, 2019). For LMICs empirical estimates are limited to two studies that each analyse a single cross-section (Ochalek et al., 2018; Woods, Revill, Sculpher, & Claxton, 2016). Nevertheless analysis of the variation between country estimates informs associations that could form the basis of forecasts. Our analysis uses a regression approach where log-transformed marginal productivity of public health care expenditure in 2015 is explained by log-transformed variables that have projected values available and categorical time-invariant variables. The estimated coefficients from this model can inform associations between proportionate changes in explanatory variables and proportionate changes in the outcome variable of interest, and how these differ by country category.

#### Outcome variable: marginal productivity of public health care expenditure ($ per DALY averted, 2017 USD)

We wish to forecast estimates of the marginal productivity of public health care expenditure for LMICs. Our analysis considers the results from Ochalek et al. (2018) and Ochalek & Lomas (2020), which – combined – give estimates of the marginal productivity of public health care expenditure for 123 countries across all income categories. Estimates for high income countries are therefore used, because we want to understand associations at higher levels of income that may be forecasted for many current LMICs. These studies provide a range of estimates for each country from which we obtain a central estimate by considering the average health consequences of a given amount of public health care expenditure. This is calculated as the inverse of the mean average of the inverted marginal productivities of public health care expenditure (denoted in terms of cost per DALY averted) estimated. This central estimate is converted into 2017 USD by applying the 2017 US GDP deflator for all countries and then log-transformed.^[[1]](#footnote-1)^

#### Explanatory variables

Our approach models the estimates from Ochalek et al. (2018) and Ochalek & Lomas (2020) as a function of variables for which we can obtain estimates of future values, either because we do not expect them to change over time or because forecasts have been made.

While a range of health spending projections have been made (Deileman et al., 2017), variables that feature directly within the studies are excluded to avoid artificial associations that exist by construction. Resulting time-varying candidate variables are GDP per capita (in 2017 USD), total fertility rate (TFR) and dependency ratio (DEP). GDP per capita (in 2017 USD) is obtained from Deileman et al. (2017) for 2015-40. Total fertility rate (live births per woman) and dependency ratio (ratio of population 0-14 and 65+ per 100 population 15-64) are obtained from United Nations Department of Economic and Social Affairs Population Division (2017) every 5 years for 2015-2100. These variables are all log-transformed prior to analysis.

Further, we consider categorizing the countries in our dataset. Income categories of countries are not considered since we already plan to analyse by GDP per capita. Instead, we categorise by considering eligibility for support from different international institutions: Global Fund (Health Systems Strengthening), IMF and GAVI. Eligibility for these support programmes is taken to reveal something about the country that does not vary over time rather than indicating an effect of eligibility itself. Alternatively we could forecast eligibility status, but it is very possible that the criteria for eligibility may themselves change in the future in ways that are hard to predict.

#### Regression approach

Associations between marginal productivity of public health care expenditure and GDP per capita (typically assuming direct proportionality) have been supposed for some time within health economics. While the rule-of-thumb that it should be 1-3x GDP per capita is now discredited by the World Health Organisation (Bertram et al., 2016), the idea has had a substantial influence on practice in cost-effectiveness analysis (Leech et al., 2018), and the search for an alternative constant multiple of GDP per capita continues (Jamison et al., 2017). For this reason we analyse the published results for 2015 with respect to GDP per capita in 2015 as our starting point. However, our analysis does not consider a linear association between the two variables, because we aim to build a model to relate proportionate changes in explanatory variables to proportionate changes in marginal productivity of public health care expenditure. As such we first estimate a linear regression model of the following form:

${\ln\left( cost per DALY averted \right)}_{i}= \beta_{0}+\beta_{1}*{\ln\left( GDP per capita \right)}_{i}+\varepsilon_{i}$ (A1)

where i denotes a given country, $\beta_{0}$ and $\beta_{1}$ are coefficients to be estimated and $\varepsilon_{i}$ is a country-specific error term. The estimated value of $\beta_{1}$ reflects the percentage change in cost per DALY averted in response to a 1% increase in GDP per capita and can be thought of as an elasticity. We assess model fit on the grounds of $R^{2}$, i.e. the proportion of variance in the outcome variable explained by the variance in explanatory variables, and through use of Ramsey RESET to indicate omitted variables and non-linearity. This regression is supplemented with additional explanatory variables where appropriate.

Within forecasting it is common to test for structural breaks over time. Assessing this is not possible in our analysis where we have only one cross-section available. Instead we analyse whether country-category structural breaks can be found in the relationship between log-marginal productivity of public health care expenditure and log-GDP per capita. This is implemented by running a linear regression with interaction terms:

${\ln\left( cost per DALY averted \right)}_{i}= \gamma_{0}+\gamma_{1}*{\ln\left( GDP per capita \right)}_{i}+\gamma_{2}*{Cat}_{i}+\gamma_{3}*{\ln\left( GDP per capita \right)}_{i}*{Cat}_{i}+\omega_{i}$ (A2)

where ${Cat}_{i}$ represents an indicator variable that is equal to 1 if the country is a member of the category and 0 otherwise. $\gamma_{0}$, $\gamma_{1}$, $\gamma_{2}$ and $\gamma_{3}$ are coefficients to be estimated and $\omega_{i}$ is a country-specific error term. A joint test of $\gamma_{2}$ and $\gamma_{3}$ can be conducted to assess the presence of a structural break by the country-category variable. In this model the cost per DALY averted elasticity of GDP per capita is given by $\gamma_{1}$ if the country is not a member of the category and $\gamma_{1}+\gamma_{3}$ of the country is a member of the category. As before, we assess model fit on the grounds of $R^{2}$ and through use of Ramsey RESET with the regression supplemented with additional explanatory variables where appropriate.

## Results

Plotting the log-transformed central estimates for marginal productivity of public health care expenditure from Ochalek et al. (2018) and Ochalek & Lomas (2020) against log-transformed GDP per capita reveals a strikingly linear relationship.

Figure A1 Scatterplot of log-transformed central estimates for marginal productivity of public health care expenditure against log-transformed GDP per capita with fitted linear plot

A simple regression of the same variables confirms what can be seen in Figure A1 with an $R^{2}$ indicating that 93% of the variation in log-transformed central estimates for marginal productivity of public health care expenditure can be explained by variation in log-transformed GDP per capita (see column 1 of Table A1). The estimated coefficient on log-transformed GDP per capita is statistically significant at the 1% significance level and suggests that a 1% increase in GDP per capita increases the cost per DALY averted by 1.4%.

Table A1 – Regression analysis of of log-transformed central estimates for marginal productivity of public health care expenditure against time-varying explanatory variables

|  | (1) | (2) | (3) | (4) | (5) |
| --- | --- | --- | --- | --- | --- |
| VARIABLES | lnCPDA | lnCPDA | lnCPDA | lnCPDA | lnCPDA |
|  |  |  |  |  |  |
| lnGDPpc | 1.400*** |  |  | 1.255*** | 1.277*** |
|  | [0.034] |  |  | [0.056] | [0.054] |
| lnDEP |  | -5.211*** |  | 0.731 |  |
|  |  | [0.521] |  | [0.471] |  |
| lnTFR |  |  | -3.756*** | -0.964*** | -0.500*** |
|  |  |  | [0.254] | [0.346] | [0.175] |
| Constant | -3.898*** | 28.957*** | 11.202*** | -4.782*** | -2.414*** |
|  | [0.286] | [2.130] | [0.263] | [1.634] | [0.590] |
|  |  |  |  |  |  |
| Observations | 123 | 122 | 122 | 122 | 122 |
| R-squared | 0.933 | 0.455 | 0.646 | 0.939 | 0.937 |
| RESET p-value | 0.0440 | 0.000905 | 0.320 | 0.0189 | 0.0151 |

Standard errors in brackets

*** p<0.01, ** p<0.05, * p<0.1

Our other time-varying explanatory variables log-transformed dependency ratio and log-transformed total fertility rate are also statistically significant at the 1% significance level when specified individually, but the $R^{2}$ of these models is much lower (columns 2 and 3 of Table A1). They are found to be negatively associated with log-transformed central estimates for marginal productivity of public health care expenditure. There is evidence that all of these regressions against the individual explanatory variables are misspecified with the exception of log-transformed total fertility rate. A modest statistically significant (at 1% level) increase in $R^{2}$ is achieved by including all three explanatory variables jointly, but a positive association is now observed for log-transformed dependency ratio that is no longer statistically significant (at 10% level, column 4 of Table A1). Log-transformed dependency ratio is dropped to obtain a more parsimonious model, in column 5, which is found to be misspecified according to RESET.

Evidence of misspecification further motivates an investigation into structural breaks between countries in terms of the relationship between log-transformed central estimates for marginal productivity of public health care expenditure and log-transformed GDP per capita. The results from our analysis are presented in Table A2.

Table A2 Regression analysis of of log-transformed central estimates for marginal productivity of public health care expenditure against time-varying explanatory variables allowing for structural breaks by country-category

|  | (1) | (2) | (3) | (4) | (5) | (6) | (7) | (8) | (9) |
| --- | --- | --- | --- | --- | --- | --- | --- | --- | --- |
| VARIABLES | lnCPDA | lnCPDA | lnCPDA | lnCPDA | lnCPDA | lnCPDA | lnCPDA | lnCPDA | lnCPDA |
|  |  |  |  |  |  |  |  |  |  |
| lnGDPpc | 1.346*** | 1.214*** | 1.248*** | 1.322*** | 1.217*** | 1.265*** | 1.409*** | 1.257*** | 1.277*** |
|  | [0.070] | [0.082] | [0.073] | [0.085] | [0.098] | [0.086] | [0.040] | [0.059] | [0.057] |
| gavi2015 | 0.701 | 1.301 | 1.813* |  |  |  |  |  |  |
|  | [0.959] | [1.130] | [0.978] |  |  |  |  |  |  |
| gavi2015_lnGDPpc | -0.134 | -0.211 | -0.274** |  |  |  |  |  |  |
|  | [0.121] | [0.142] | [0.124] |  |  |  |  |  |  |
| lnTFR |  | -0.892** | -0.617*** |  | -0.854** | -0.531*** |  | -0.960*** | -0.574*** |
|  |  | [0.353] | [0.180] |  | [0.366] | [0.183] |  | [0.348] | [0.182] |
| lnDEP |  | 0.483 |  |  | 0.563 |  |  | 0.627 |  |
|  |  | [0.532] |  |  | [0.552] |  |  | [0.481] |  |
| GFHSS2015 |  |  |  | -0.105 | 0.215 | 0.835 |  |  |  |
|  |  |  |  | [0.984] | [1.184] | [1.016] |  |  |  |
| GFHSS2015_lnGDPpc |  |  |  | -0.026 | -0.057 | -0.130 |  |  |  |
|  |  |  |  | [0.113] | [0.136] | [0.116] |  |  |  |
| imf2015 |  |  |  |  |  |  | 0.543 | 1.045 | 1.264 |
|  |  |  |  |  |  |  | [0.868] | [0.885] | [0.871] |
| imf2015_lnGDPpc |  |  |  |  |  |  | -0.074 | -0.146 | -0.175 |
|  |  |  |  |  |  |  | [0.117] | [0.120] | [0.118] |
| Constant | -3.342*** | -3.410** | -2.012*** | -3.088*** | -3.770** | -2.222** | -3.976*** | -4.381*** | -2.353*** |
|  | [0.653] | [1.711] | [0.740] | [0.818] | [1.742] | [0.856] | [0.346] | [1.671] | [0.614] |
|  |  |  |  |  |  |  |  |  |  |
| Observations | 123 | 122 | 122 | 123 | 122 | 122 | 123 | 122 | 122 |
| R-squared | 0.936 | 0.942 | 0.942 | 0.935 | 0.940 | 0.940 | 0.933 | 0.939 | 0.938 |
| RESET p-value | 0.112 | 0.111 | 0.199 | 0.0716 | 0.0236 | 0.0602 | 0.0472 | 0.0383 | 0.0511 |
| Structural break test p-value | 0.0604 | 0.0262 | 0.0118 | 0.120 | 0.208 | 0.106 | 0.821 | 0.473 | 0.337 |

Standard errors in brackets

*** p<0.01, ** p<0.05, * p<0.1

Results for a model allowing for a structural break between GAVI-eligible countries and others in terms of the relationship between the outcome variable and log-transformed GDP per capita is shown in column 1 of Table A2 with the p-value in the bottom row indicating rejection of the test of the null hypothesis of no structural break at the 10% significance level. This is illustrated in Figure A2 where the GAVI-eligible countries are indicated with a red dot.

Figure A2 Scatterplot of log-transformed central estimates for marginal productivity of public health care expenditure against log-transformed GDP per capita allowing for structural break by GAVI eligibility with fitted linear plots

Structural breaks by the other country-category variables, Global Fund-eligible and IMF-eligible, provide little evidence against the null hypothesis of no break (columns 4 and 7 of Table A2). All models allowing a structural break are supplemented with additional explanatory variables (columns 2, 5 and 8), which are dropped from the specification if they are found to not be statistically significant in the interest of a parsimonious model (columns 3, 6 and 9).

Our preferred model for log-transformed central estimates for marginal productivity of public health care expenditure is given in column 3 of Table A2, which allows for a structural break in the relationship with log-transformed GDP per capita by GAVI-eligibility and controls for log-transformed total fertility rate. This model explains 94.2% of the variance in the outcome variable and does not appear to be misspecified according to the RESET. The model indicates that marginal productivity of public health care expenditure increases by 1.2% with a 1% increase in GDP per capita for non-GAVI-eligible countries, but by only 1% for GAVI-eligible countries. In addition, a 1% decrease in total fertility rate is associated with a 0.6% increase in marginal productivity of public health care expenditure. With this satisfactory econometric model specified for 2015 values, we apply the coefficient estimates to projections of the explanatory variables in order to forecast marginal productivity of public health care expenditure for each country for 2016-2040 for use in our illustrative example (where members of this country-category are assumed to remain the same over time).

The results from this model are presented in the Figure A3 where each country is represented by a different colour line and the trends over time can be observed. From this Figure it can be seen that marginal productivity of public health care expenditure is projected to increase in real terms for most countries, but that there are considerable differences By way of summary, the average results for each country are presented in Table A3, which contains the GAVI status and compound annual growth rate over the period between 2015 and 2040 of GDP per capita, total fertility rate and marginal productivity of public health care expenditure. The full results for each country, i.e. for each time period, are presented in Appendix B along with values for all other evaluation parameters.

Figure A3 Projected marginal productivity of public health care expenditure by country 2015-2040 (2015 = 100)

Table A3 – Summary results for projected changes in marginal productivity of public health care expenditure

|  | | | CAGR between 2015 and 2040 | | |
| --- | --- | --- | --- | --- | --- |
| Country | Code | GAVI | GDP per capita | TFR | k |
| Albania | ALB | 0 | 2.9% | 0.0% | 3.4% |
| Algeria | DZA | 0 | 0.8% | -1.1% | 1.4% |
| Argentina | ARG | 0 | 0.7% | -0.6% | 1.1% |
| Armenia | ARM | 1 | 1.9% | 0.1% | 1.9% |
| Azerbaijan | AZE | 1 | 0.9% | -0.4% | 1.0% |
| Bangladesh | BGD | 1 | 4.6% | -0.8% | 4.7% |
| Belarus | BLR | 0 | 0.5% | 0.3% | 0.5% |
| Belize | BLZ | 0 | 0.5% | -0.9% | 1.1% |
| Benin | BEN | 1 | 1.9% | -1.3% | 2.3% |
| Bolivia | BOL | 1 | 2.4% | -1.0% | 2.6% |
| Botswana | BWA | 0 | 2.0% | -1.1% | 2.7% |
| Brazil | BRA | 0 | 0.3% | -0.2% | 0.5% |
| Bulgaria | BGR | 0 | 2.6% | 0.4% | 2.9% |
| Burkina Faso | BFA | 1 | 3.5% | -1.5% | 3.8% |
| Burundi | BDI | 1 | -0.3% | -1.5% | 0.4% |
| Cambodia | KHM | 1 | 4.2% | -0.9% | 4.3% |
| Cameroon | CMR | 1 | 2.3% | -1.3% | 2.6% |
| Cape Verde | CPV | 0 | 1.7% | -1.0% | 2.3% |
| Chad | TCD | 1 | -0.2% | -1.7% | 0.6% |
| China | CHN | 0 | 5.3% | 0.2% | 6.0% |
| Colombia | COL | 0 | 1.3% | -0.4% | 1.7% |
| Comoros | COM | 1 | 0.0% | -1.3% | 0.7% |
| Congo | COG | 1 | -0.5% | -1.2% | 0.2% |
| Costa Rica | CRI | 0 | 2.4% | -0.2% | 2.9% |
| Cote d'Ivoire | CIV | 1 | 2.0% | -1.1% | 2.4% |
| Democratic Republic of the Congo | COD | 1 | 0.1% | -1.9% | 0.9% |
| Dominican Republic | DOM | 0 | 3.4% | -0.9% | 4.2% |
| Ecuador | ECU | 0 | 0.5% | -0.9% | 1.0% |
| Egypt | EGY | 0 | 1.9% | -1.0% | 2.6% |
| El Salvador | SLV | 0 | 1.6% | -0.7% | 2.2% |
| Ethiopia | ETH | 1 | 5.1% | -2.0% | 5.3% |
| Gabon | GAB | 0 | 0.4% | -1.3% | 1.0% |
| Georgia | GEO | 1 | 2.0% | -0.2% | 2.0% |
| Ghana | GHA | 1 | 3.1% | -1.2% | 3.3% |
| Guatemala | GTM | 0 | 1.2% | -1.2% | 1.8% |
| Guinea | GIN | 1 | 2.4% | -1.5% | 2.8% |
| Guinea-Bissau | GNB | 1 | 1.6% | -1.4% | 2.0% |
| Guyana | GUY | 1 | 2.5% | -0.8% | 2.7% |
| Haiti | HTI | 1 | 0.2% | -1.2% | 0.8% |
| Honduras | HND | 1 | 1.7% | -1.0% | 2.0% |
| India | IND | 1 | 4.9% | -0.8% | 5.0% |
| Indonesia | IDN | 1 | 4.1% | -0.7% | 4.2% |
| Jamaica | JAM | 0 | 0.5% | -0.5% | 0.8% |
| Jordan | JOR | 0 | 0.9% | -1.4% | 1.6% |
| Kazakhstan | KAZ | 0 | 1.6% | -0.7% | 2.2% |
| Kenya | KEN | 1 | 1.8% | -1.3% | 2.2% |
| Kyrgyzstan | KGZ | 1 | 0.8% | -0.9% | 1.2% |
| Lebanon | LBN | 0 | 0.6% | 0.0% | 0.7% |
| Lesotho | LSO | 1 | 2.9% | -1.1% | 3.1% |
| Macedonia | MKD | 0 | 2.0% | 0.3% | 2.3% |
| Madagascar | MDG | 1 | 1.3% | -1.2% | 1.7% |
| Malawi | MWI | 1 | 0.4% | -1.4% | 1.0% |
| Malaysia | MYS | 0 | 2.3% | -0.5% | 2.9% |
| Mali | MLI | 1 | 2.3% | -1.8% | 2.8% |
| Mauritania | MRT | 1 | 0.6% | -1.2% | 1.1% |
| Mauritius | MUS | 0 | 3.2% | 0.4% | 3.6% |
| Mexico | MEX | 0 | 1.0% | -0.8% | 1.6% |
| Moldova | MDA | 1 | 1.5% | 0.8% | 1.1% |
| Mongolia | MNG | 1 | 1.6% | -0.8% | 1.9% |
| Morocco | MAR | 0 | 2.6% | -0.9% | 3.3% |
| Mozambique | MOZ | 1 | 2.1% | -1.3% | 2.4% |
| Namibia | NAM | 0 | 0.7% | -1.2% | 1.3% |
| Nepal | NPL | 1 | 2.7% | -0.8% | 2.9% |
| Nicaragua | NIC | 1 | 2.1% | -0.8% | 2.3% |
| Niger | NER | 1 | 1.1% | -1.3% | 1.6% |
| Nigeria | NGA | 1 | 0.0% | -1.4% | 0.6% |
| Pakistan | PAK | 1 | 2.7% | -1.3% | 3.0% |
| Panama | PAN | 0 | 3.5% | -0.8% | 4.2% |
| Paraguay | PRY | 0 | 2.5% | -0.8% | 3.2% |
| Peru | PER | 0 | 2.2% | -0.9% | 2.8% |
| Philippines | PHL | 0 | 3.6% | -0.9% | 4.3% |
| Romania | ROU | 0 | 3.3% | 0.4% | 3.7% |
| Russia | RUS | 0 | 0.5% | 0.3% | 0.5% |
| Rwanda | RWA | 1 | 4.0% | -1.6% | 4.3% |
| Senegal | SEN | 1 | 2.8% | -1.2% | 3.0% |
| Sierra Leone | SLE | 1 | 0.8% | -1.9% | 1.5% |
| South Africa | ZAF | 0 | 0.1% | -0.8% | 0.5% |
| Sri Lanka | LKA | 1 | 4.0% | -0.5% | 4.0% |
| Sudan | SDN | 1 | 1.2% | -1.2% | 1.7% |
| Swaziland | SWZ | 0 | 0.6% | -1.2% | 1.3% |
| Tajikistan | TJK | 1 | 1.3% | -1.0% | 1.6% |
| Tanzania | TZA | 1 | 3.5% | -1.2% | 3.7% |
| Thailand | THA | 0 | 2.3% | 0.3% | 2.7% |
| The Gambia | GMB | 1 | 0.3% | -1.7% | 1.0% |
| Togo | TGO | 1 | 1.8% | -1.2% | 2.1% |
| Tunisia | TUN | 0 | 0.7% | -0.5% | 1.1% |
| Turkey | TUR | 0 | 3.1% | -0.5% | 3.8% |
| Turkmenistan | TKM | 0 | 3.7% | -1.0% | 4.4% |
| Uganda | UGA | 1 | 1.4% | -1.6% | 1.9% |
| Ukraine | UKR | 1 | -0.2% | 0.4% | -0.5% |
| Uzbekistan | UZB | 1 | 2.8% | -0.7% | 3.0% |
| Vietnam | VNM | 1 | 4.8% | -0.1% | 4.7% |
| Yemen | YEM | 1 | -0.2% | -1.9% | 0.7% |
| Zambia | ZMB | 1 | 1.2% | -1.1% | 1.6% |
| Zimbabwe | ZWE | 1 | -0.1% | -1.4% | 0.6% |

1. <https://data.worldbank.org/indicator/NY.GDP.DEFL.ZS?locations=US> [↑](#footnote-ref-1)
